# Supplementary material for: A Kinematic Deviation Index (KDI) for Evaluation of Forelimb Function in Rodents
Source: bioRxiv. 2024 Sep 29:2024.09.26.615237. Preprint. [Version 1] doi: 10.1101/2024.09.26.615237 (PMC11463371; doi:10.1101/2024.09.26.615237)
Supplement: 1 [file NIHPP2024.09.26.615237V1-supplement-1.pdf]

bioRxiv preprint doi: https://doi.org/10.1101/2024.09.26.615237; this version posted September 29, 2024. The copyright holder for this preprint (which was not certified by peer review) is the author/funder, who has granted bioRxiv a license to display the preprint in perpetuity. It is made available under aCC-BY 4.0 International license.

Supplementary Figures

bioRxiv preprint doi: https://doi.org/10.1101/2024.09.26.615237; this version posted September 29, 2024. The copyright holder for this preprint (which was not certified by peer review) is the author/funder, who has granted bioRxiv a license to display the preprint in perpetuity. It is made available under aCC-BY 4.0 International license.

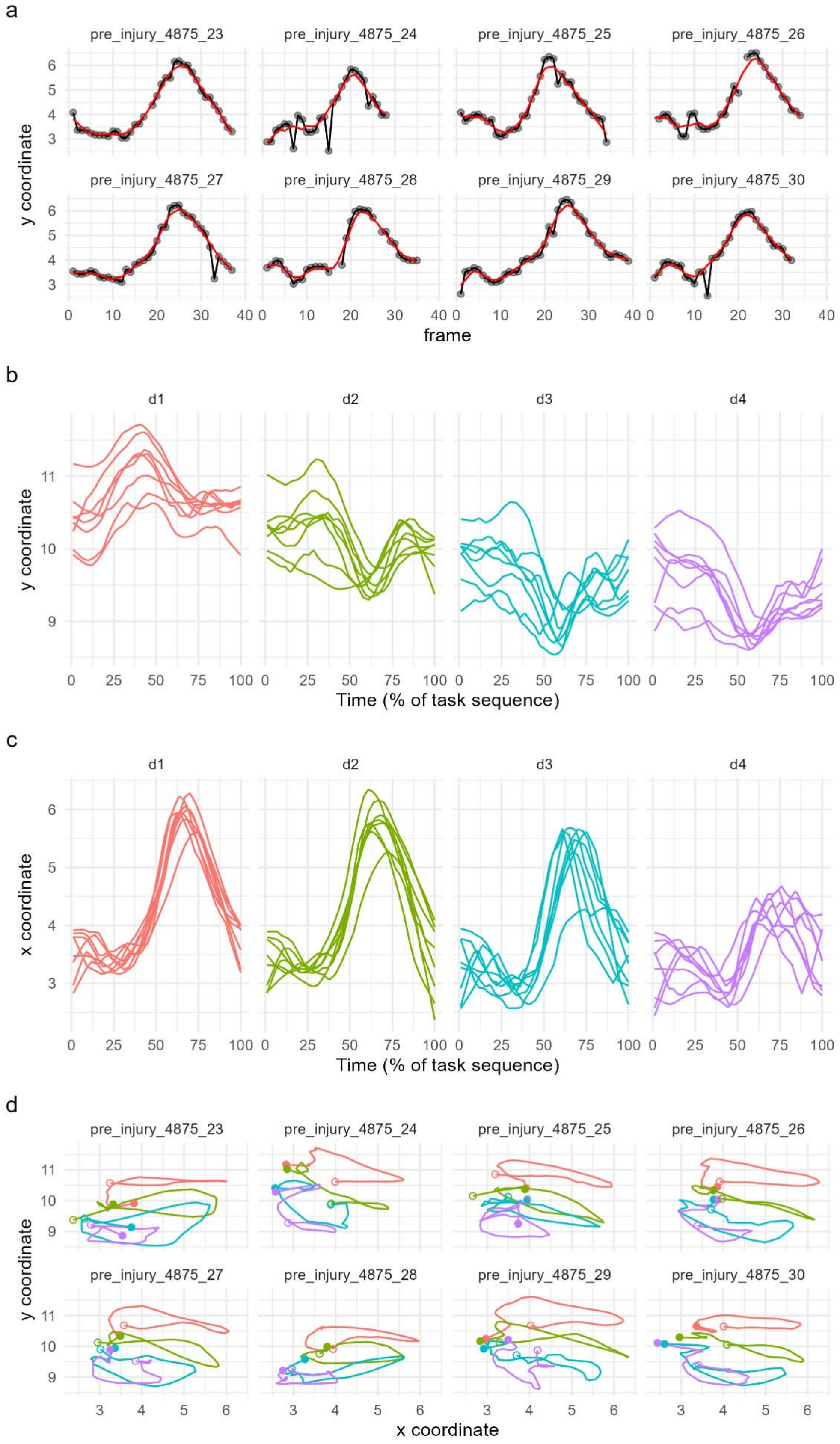

bioRxiv preprint doi: https://doi.org/10.1101/2024.09.26.615237; this version posted September 29, 2024. The copyright holder for this preprint (which was not certified by peer review) is the author/funder, who has granted bioRxiv a license to display the preprint in perpetuity. It is made available under aCC-BY 4.0 International license.

Supplementary Figure 1. Example of the processing of the raw kinematic data for a set of trials from the same animal. (a) illustration of the denoising and smoothing of the y coordinate of the marker for d1 marker for the successful trials of the same animal from the first cohort pre-injury. The dots and black lines represent the original raw trajectory over the frame sequence, and the red line is the result of denoising and smoothing to consider spatiotemporal coherence. (b) The y coordinate of the time-normalized sequence for all four-digit markers of the same animal. (c) The x coordinate of the time-normalized sequence for all four-digit markers for the same animal. (d) The xy coordinate trajectories for the four-digit markers for the same animal. Close dots show the start of the sequence, and open dots show the end. Colors denote the different digits.
